# A review of the biology and therapeutic implications of cancer-associated fibroblasts (CAFs) in muscle-invasive bladder cancer

**DOI:** 10.3389/fonc.2022.1000888

**Published:** 2022-10-13

**Authors:** Amy Burley, Antonio Rullan, Anna Wilkins

**Affiliations:** ^1^ Division of Radiotherapy and Imaging, Institute of Cancer Research, London, United Kingdom; ^2^ Head and Neck Unit, Royal Marsden National Health Service (NHS) Hospital Trust, London, United Kingdom; ^3^ Department of Radiotherapy, Royal Marsden National Health Service (NHS) Hospital Trust, London, United Kingdom

**Keywords:** cancer-associated fibroblast, muscle-invasive bladder cancer, bladder cancer, caf, immunotherapy, radiotherapy

## Abstract

Cancer-associated fibroblasts (CAFs) play a fundamental role in the development of cancers and their response to therapy. In recent years, CAFs have returned to the spotlight as researchers work to unpick the mechanisms by which they impact tumour evolution and therapy responses. However, study of CAFs has largely been restricted to a select number of common cancers, whereas research into CAF biology in bladder cancer has been relatively neglected. In this review, we explore the basics of CAF biology including the numerous potential cellular origins of CAFs, alongside mechanisms of CAF activation and their diverse functionality. We find CAFs play an important role in the progression of bladder cancer with significant implications on tumour cell signaling, epithelial to mesenchymal transition and the capacity to modify components of the immune system. In addition, we highlight some of the landmark papers describing CAF heterogeneity and find trends in the literature to suggest that the iCAF and myCAF subtypes defined in bladder cancer share common characteristics with CAF subtypes described in other settings such as breast and pancreatic cancer. Moreover, based on findings in other common cancers we identify key therapeutic challenges associated with CAFs, such as the lack of specific CAF markers, the paucity of research into bladder-specific CAFs and their relationship with therapies such as radiotherapy. Of relevance, we describe a variety of strategies used to target CAFs in several common cancers, paying particular attention to TGFβ signaling as a prominent regulator of CAF activation. In doing so, we find parallels with bladder cancer that suggest CAF targeting may advance therapeutic options in this setting and improve the current poor survival outcomes in bladder cancer which sadly remain largely unchanged over recent decades.

## 1 Introduction

### 1.1 Brief overview of bladder cancer

Approximately 20,000 people are diagnosed with bladder cancer each year in the UK (1). Typically, patients are aged 75 years or greater at diagnosis and are predominantly male. However, bladder cancer affects all genders and also occurs in younger patients, and the most clearly established risk factor for bladder cancer is smoking (2). In the past 30 to 40 years, there have been minimal advances in the survival outcomes of patients diagnosed with bladder cancer. Indeed, the 5-year survival following radical cystectomy or chemo-radiation remains at approximately 50% (3). Therefore, there is an urgent need to better understand bladder cancer biology to help find more effective curative treatments.

**Figure 1:**
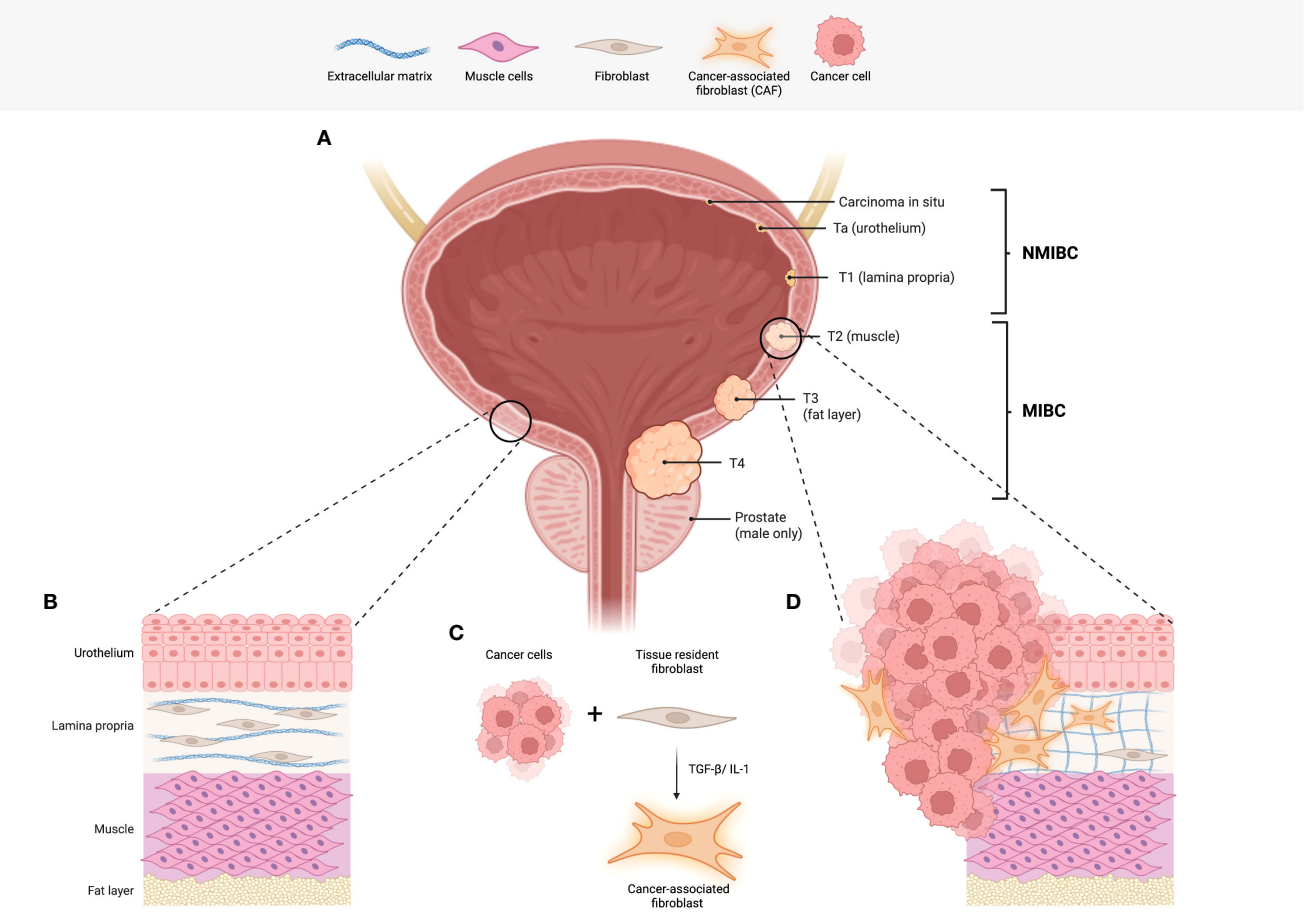
Bladder cancer staging and the role of fibroblasts. **(A)** An illustration of the human bladder, including the tumour (T stages) of bladder cancer. Commencing in the urothelium, NMIBCs describe carcinoma *in situ* (in the inner most layer of the urothelium) Ta (urothelium only) and T1 tumours. T1 tumours have infiltrated the first sub-urothelial layer known as the lamina propria. To progress to stages T2-4, invasion into the muscle layer is required and is the determining factor in the diagnosis of MIBC. **(B)** A representation of the physiological histology observed in the human bladder. Of note, fibroblasts found in the lamina propria function to produce and maintain collagen and other fibres that make up the loose connective tissue. **(C)** Interactions between tumour cells and tissue resident fibroblasts are one of the likely origins of cancer-associated fibroblasts (CAFs). **(D)** The illustration depicts the histological changes observed in bladder cancer including the presence of CAFs. In addition, there is an increase in extracellular matrix deposition and remodelling. Abbreviations: non-muscle invasive bladder cancer (NMIBC), muscle invasive bladder cancer (MIBC), cancer-associated fibroblasts (CAFs). Figure created with Biorender.com.

The most common form of bladder cancer arises from cells in the bladder lining, or urothelium, and is consequently known as urothelial bladder carcinoma. The anatomy of the bladder and stages of bladder cancer, defined according to how far the cancer cells have invaded into the bladder wall, are shown in **Figure 1**. Early stage, non-muscle-invasive bladder cancers (NMIBC) reside within the bladder lining and are risk-stratified depending on the tumour grade, a measure of tumour cell growth rate. In contrast, muscle-invasive bladder cancer (MIBC) describes tumours that have grown into the muscle wall of the bladder, referred to as the muscularis propria.

Comprehensive analysis of gene expression data resulted in the molecular classification of MIBC and the identification of molecular subtypes by several research groups (4–9). In a recent consensus classification, six subtypes have been defined: luminal papillary, luminal non-specified, luminal unstable, stroma-rich, basal/squamous, and neuroendocrine-like (10). The molecular classification of MIBC has helped to increase knowledge of bladder cancer and find potential associations between subtypes, clinical characteristics, response to therapies, and survival. While the neuroendocrine-like subtype is associated with the worst prognosis, it is interesting to note that the stroma-rich subtype appears to have a particularly poor response to neo-adjuvant chemotherapy (10). Moreover, the stroma-rich subtype has an over-expression of gene signatures associated with smooth muscle, endothelial cells, fibroblasts and myofibroblasts (10).

### 1.2 Treatment of NMIBC vs MIBC

Currently, all patients with bladder cancer undergo a biopsy-like procedure known as a transurethral resection of bladder tumour (TURBT) which acts as both a diagnostic tool to clarify the presence or absence of tumour cell invasion into muscle and a debulking procedure to remove as much of the visible tumour mass as possible. In patients with high grade NMIBC, immunotherapeutic agents such as Bacillus Calmette–Guérin (BCG) are commonly used after TURBT; BCG works to stimulate an anti-tumour immune response to tackle residual cancer cells. Intriguingly, BCG remains one of the longest-established immunotherapies ever used in cancer treatment.

If muscle invasion is identified during the pathological assessment of the TURBT tissue, MIBC patients are treated surgically with a cystectomy or bladder-sparing treatments (BST) such as radiotherapy. Following the improved locoregional control seen with the addition of concomitant 5-fluorouracil and mitomycin C to radiotherapy in the phase III UK BC2001 trial, led by James et al., provision of BST typically includes radiotherapy in combination with chemotherapy (11). This combinatorial approach can offer quality of life benefits in selected patients and can also be a useful option for patients who are unable to undergo cystectomy (11, 12). Of relevance, following BST, patients will require lifelong cystoscopic surveillance due to the risk of recurrence (13), alongside the cross-sectional imaging and clinical surveillance carried out following treatment in all patients.

### 1.3 Research challenges and scope of this review

At present, we lack predictive biomarkers to identify which patients may be more likely to relapse following BST and could therefore be better surgical candidates or require treatment intensification. A strong predictive biomarker is therefore a key unmet clinical need to aid radical treatment decisions.

Recently, stromal cell populations in the tumour microenvironment of solid tumours, known as cancer-associated fibroblasts (CAFs), have gained prominence as an important cell type influencing bladder cancer survival outcomes.

In metastatic bladder cancer, poor responses to immune checkpoint inhibitors have been associated with TGFβ signaling in fibroblasts (14) and high EMT/stromal related gene expression (15). Moreover, in cases with poor responses, CD8 T-cells are frequently found trapped in the peri-tumoural regions amongst fibroblasts and collagen-rich matrix (14). High EMT/stromal gene expression and low tumour-infiltrating T cell abundance have both also been associated with inferior outcomes following cystectomy (15). Unfortunately, research into the effect of CAFs on radiotherapy responses, and, similarly, the impact of radiotherapy on CAF biology, is in its infancy and warrants further investigation in the setting of bladder cancer.

In this review we will outline the latest consensus views on CAF biology with a specific focus on bladder cancer. We will introduce some of the current controversies regarding CAF heterogeneity and disease-specific CAF subtypes. This review will discuss the gaps in our current understanding of the biological relevance of CAFs in bladder cancer – here, we will draw from studies of CAFs in other cancers to explore longitudinal changes in CAF biology during therapy. Finally, we will discuss whether the addition of therapeutic approaches to specifically target CAFs may improve survival in bladder cancer.

## 2 The tumour microenvironment and the role of CAFs

The tumour microenvironment (TME) describes the ecosystem formed by many different cell populations coexisting within the tumour. Within the TME, CAFs help to create the structural framework, including the extracellular matrix (ECM), within which all other cells in the TME reside. Such cells include, but are not limited to, cancer cells, cytotoxic and regulatory immune cells, antigen-presenting cells and cells of the vasculature including endothelial cells.

### 2.1 Origins and activation of CAFs

#### 2.1.1 Origins

CAFs are often classified as non-neoplastic, not epithelial, endothelial, or immune cells. As opposed to normal fibroblasts that can be temporarily activated, CAFs are persistently activated, usually *via* epigenetic reprogramming (16).

CAFs are derived from several potential sources: the most commonly cited and likely origin is from normal fibroblasts resident in the tissue of tumour origin that have undergone activation *via* processes outlined below (17) (see **Figure 1**); secondly, CAFs may be mesenchymal stem cells derived from the bone marrow (18). Some anecdotal studies suggest that CAFs can arise from cells of the vasculature such as endothelial cells, as well as pericytes and adipocytes (19). Furthermore, the specific cell of origin may be indicative of the CAFs eventual function and role in the TME (20). A fascinating recent study utilised single cell RNA sequencing (scRNAseq) data atlases for healthy and disease state tissues of human and mouse derivation to trace the lineage of fibroblasts (21). In doing so, they suggest that all fibroblasts, whether healthy, tissue-specific, or activated in disease, may all share a common ancestor. From such an ancestor, tissue-specific functions are subsequently acquired *in situ*, typically *via* changes to their epigenetic and transcriptional profile (21).

#### 2.1.2 Activation

In addition to their cellular origin, the mechanism by which CAFs are activated seemingly imparts an additional layer of information that can provide functional and spatial stratification of CAFs. Activation of CAFs by cancer cells has been explored in several studies. For example, using *in vitro* and *in vivo* experiments, Strell et al. showed direct cell to cell contact between ductal breast carcinoma *in situ* (DCIS) cancer cells and fibroblasts induces changes in the expression profile of fibroblasts, eventually resulting in a platelet-derived growth factor (PDGF) receptor (PDGFR)α low, PDGFRβ high subset of CAFs (22). Interestingly, assessment of PDGFRα and PDGFRβ expression in tissue specimens from 458 primary DCIS patients showed an increased proportion of the PDGFRα low PDGFRβ high CAFs was associated with a higher risk of recurrence (22).

In addition to the direct cell contact driving activation of CAFs described above, paracrine crosstalk between cancer cells and fibroblasts can also lead to acquisition of the CAF phenotype. *In vitro* studies of bladder cancer cell lines revealed a high content of interleukin (IL)-1α in tumour conditioned media (CM) (23). When fibroblasts were cultured in IL-1α rich CM, this led to the release of cytokines and pro-tumour factors such as IL-8, granulocyte-macrophage colony-stimulating factor (GM-CSF), monocyte chemoattractant protein-1 (MCP-1) and hepatocyte growth factor (HGF), i.e. acquisition of an “activated CAF phenotype” which subsequently promoted cancer cell migration in Boyden chamber assays (23).

In a further study, Öhlund et al. demonstrated that both cell to cell contact and paracrine crosstalk between cancer cells and CAFs occur in distinct regions of pancreatic tumours with differing results (24). Contact between fibroblast activation protein (FAP)+ pancreatic stellate cells (PSC) and pancreatic cancer cells resulted in activation and conversion of PSC into CAFs, represented by an increase in expression of alpha smooth muscle actin (αSMA). These CAFs were subsequently classified as myofibroblastic CAFs (myCAFs) with the ability to deposit components of the ECM. In contrast, PSCs with less proximity to cancer cells were activated in a paracrine manner resulting in a reduced expression of αSMA and an increase in the secretion of IL-6; these CAFs were designated as inflammatory CAFs (iCAFs). Under differing conditions, CAFs appear to have the capacity to be directed into either myCAFs or iCAFs by modifying their expression profiles, suggesting that different, spatially distinct CAF subtypes exist, with functional characteristics dependent on activation (24). Of further interest, using scRNAseq, the same group identified an additional CD74+ MHC class II+ CAF subtype, denoted as antigen-presenting CAFs (apCAF) (17).

Studying single cell sequencing outputs of fibroblasts extracted from normal and pancreatic ductal adenocarcinoma (PDAC) mouse models, Dominguez et al. developed an evolutionary model to characterise CAF heterogeneity (25). Using dimensionality reduction and pseudotime analysis, they demonstrated that the previously described iCAF and myCAF populations were derived from a common early CAF subtype (25). This supported earlier work that showed mechanistically how signalling *via* IL-1 and transforming growth factor beta (TGFβ) drives the differentiation of iCAFs and myCAFs, respectively, from a common “early CAF” ancestor (26). The diversity of CAF subtypes is of considerable biological importance, and we will return to this concept in greater detail below.

### 2.2 Functions of CAFs

Pathologically, CAFs exhibit a wide range of functions that can modify the TME *via* diverse non-immune and immune mechanisms. This section will explore the functions of CAFs with reference to both bladder cancer and wider tumour biology. To help understand pathological CAF function, we will first describe the role of physiologically normal fibroblasts in the bladder: Such fibroblasts are situated in the sub-urothelial lamina propria layer of the bladder where their function includes the production of extracellular matrix components such as collagen and fibronectin (27). Uniquely, in the bladder, myofibroblasts help to transmit signals from the urothelium in response to bladder filling or pathological stressors (28). As discussed earlier, these normal fibroblasts are likely to be an important source of persistently activated CAFs with functions as outlined below:

#### 2.2.1 Production and remodeling of the ECM

One of the key functions of CAFs is to deposit and modify the filamentous ECM which facilitates tumour cell invasion (29). This has several biomechanical consequences including the generation of greater contractile strength and tension in the tissue and increased tumour stiffness. Such increased stiffness can result in further activation of CAFs, thus creating a self-perpetuating positive feedback loop. This loop is thought to arise following the activation of transcription factor Yes-associated protein (YAP) which subsequently regulates the downstream expression of genes associated with cytoskeletal and matrix remodeling (29). The increased stiffness can also impact oncogenic signalling within the tumour cell, including *via* the mitogen-activated protein kinase (MAPK) pathway (30).

#### 2.2.2 Impact on tumour cell signalling

CAFs have been shown to directly impact the proliferation and invasiveness of bladder cancer cells *via* several mechanisms including the exosomal transfer of non-coding RNA fragments such as LINC00355 (31). Components of the CAF secretome, such as microfibrillar-associated protein 5 (MFAP5), have a similar pro-tumour effect (32). Additionally, conducting co-culture studies of CAFs with bladder cancer cell lines showed that cancer cell proliferation and invasion was enhanced in conditions where CAFs were induced to undergo autophagy (33).

Further *in vitro* studies support the proposed pro-tumour impact of CAFs on bladder cancer cells. One such study showed that CAFs, but not normal fibroblasts, secrete pro-tumour cytokines such as IL-1β which activates the Wnt signalling pathway in bladder cancer cell lines. Indeed, inhibition of Wnt signalling abolished this pro-tumour effect (34). Similar effects were seen following the exosomal transfer of miR-148b-3p which induced increased invasive behaviour of bladder cancer cells *via* Wnt signalling, an effect which could be attenuated *via* the upregulation of phosphatase and tensin homolog (PTEN) (35). Overall, this suggests CAFs may impart a pro-tumour effect in bladder cancer *via* Wnt signalling pathway activation.

In addition to the pro-tumour signaling described above, CAFs have also been shown to contribute to metabolic reprogramming of tumours (36). As with the Warburg effect witnessed in cancer cells, CAFs are capable of upregulating components of the glycolytic pathway leading to an upregulation in lactate production (37). Moreover, exposure to CAF conditioned media has been shown to enhance the glycolytic activity of pancreatic cancer cells (38) helping to support the metabolic needs of surrounding cancer cells.

#### 2.2.3 Induction of epithelial to mesenchymal transition

CAFs can also have a pro-tumour effect *via* the induction of epithelial to mesenchymal transition (EMT). Indeed, secretion of IL-6 by tumour-activated CAFs was shown to induce EMT in bladder cancer cell lines (39). Here, observations included the upregulation of proteins associated with EMT such as N-cadherin and vimentin and an increase in EMT-inducing transcription factors SNAIL, TWIST and ZEB1 in bladder cancer cell lines (39). These findings are further supported by immunohistochemistry analysis of urinary bladder carcinoma which revealed the upregulation of several CAF markers was associated with an increase in markers of EMT on multiple cell types within the TME (40).

#### 2.2.4 Immunomodulatory functions of CAFs

CAFs have multiple immunomodulatory functions, which are generally, but not exclusively, considered to be immunosuppressive in nature. Such functions can be driven by the *physical* properties of CAFs for example the sequestration or trapping of immune cells in the matrix or by *secretion* of immunomodulatory chemokines for example TGFβ (41, 42). Obviously, both physical and secretory immunomodulatory functions may co-exist within a single tumour.

In a landmark paper published in 2018, Mariathasan et al. were one of the first authors to describe the role of CAFs in the creation of an immune excluded phenotype, and the associated lack of response to anti-programmed death-ligand 1 (PD-L1) checkpoint inhibitor, atezolizumab (14). Of note, this finding was demonstrated in the setting of metastatic urothelial bladder carcinoma and therefore highlights the fundamental role CAFs play in this disease (14). We will explore additional findings from this paper in subsequent sections. Below we have detailed additional studies which have explored the interplay between CAFs and cells of the immune system.

In bladder cancer, FAP+ CAFs have been associated with immune cold TMEs with poor infiltration of CD8+ T-cells and with considerable loss of human leukocyte antigen (HLA-I) expression on tumour cells (43). Poor CD8+ T cell infiltration is also likely to be due to the deposition of a heavily crosslinked ECM which acts as a physical barrier to exclude lymphocytes in peritumour regions, preventing them from reaching the tumours cells and unleashing their full cytotoxic potential (44).

To note, CAFs impact many immune populations other than lymphocytes. For example, in lung squamous cell carcinoma, CAFs have been associated with a higher infiltrate of immunosuppressive cells such as tumour-associated macrophages (45).

Although the immunomodulatory effects of CAFs are predominantly immunosuppressive and tumour-promoting, in melanoma a podoplanin (PDPN)+, FAP- CAF subtype has been shown to act in an immunostimulatory manner *via* development of tumour-associated tertiary lymphoid structures (TA-TLS). In this setting, an increase of TA-TLS was positively correlated with improved patient survival and response to immune checkpoint therapies (46). Tertiary lymphoid structures (TLS) are equally relevant in bladder cancer. In a study of immune checkpoint inhibitors targeting PD-L1 and CTLA-4, Gao et al. report the enhanced treatment response associated with a high density of TLS in the pre-treatment biopsies of patients with high grade urothelial carcinoma (47). It would be very interesting to determine the phenotype of CAFs in these patients, including the CAFs specifically present in the TLS.

Considering the diversity in the functions of CAFs described above, it is of great importance that we correctly characterise CAF subtypes and consider any implications that may follow depletion of some or all CAF populations.

### 2.3 CAF heterogeneity

Some markers such as FAP and αSMA are used frequently in CAF research and are often considered to be “canonical”. Using individual canonical CAF markers, many studies have attempted to identify the total CAF population leading to conflicting results - this is likely to be due to the heterogeneous nature of CAFs. Indeed, at present there is no known single marker that is capable of identifying all CAF populations or of segregating them from other stromal populations.

To explore CAF heterogeneity and characterise distinct CAF subtypes a variety of methods have been used across multiple cancer types, including scRNASeq, spatial transcriptomics, flow cytometry and immunohistochemistry. Each method offers a unique opportunity to study the underlying biology but can come with disadvantages. Briefly, bulk RNA sequencing facilitates a broad overview of gene signatures and signalling pathways that may be up or down regulated in the transcriptome but lacks the single cell resolution that is provided by scRNAseq. Protein expression can be studied at single cell resolution using flow cytometry, however spatial information is lost in all three of these methods. Techniques such as multiplex immunofluorescence and imaging mass cytometry help to overcome these challenges and provide insights into the interactions between different cell types. However, these techniques come with time and cost limitations that can restrict high throughput analysis.

Across many studies, the concept of CAF heterogeneity, i.e. different CAF subtypes in a single tumour, generally holds true and typically shows the distinction between iCAFs and myCAFs described above. However, beyond this, subtype classification, descriptions of functionality and biomarker selection are inconsistent and vary greatly depending on the method of CAF profiling, tumour sample quality and disease type. The type of biopsy used to collect tissue and thus stratify CAFs can also have implications on the interpretations of results. In bladder cancer, a TURBT may only provide access to superficial regions of tumour and thus not reveal the full extent of the CAF infiltrated tumour layers, resulting in a loss of heterogeneity. In contrast, a full cystectomy will typically provide access to the whole tumour and surrounding healthy tissue. These limitations surrounding inter and intra tumoural heterogeneity are not restricted to bladder cancer; the renal and lung TRACERx studies (48–50) have demonstrated the importance of evaluation of biopsies from multiple different sites within a tumour to comprehensively profile cancer biology, rather than a single snapshot.

To study inter-tissue CAF heterogeneity, Galbo et al. utilised scRNAseq data from melanoma, head and neck, and lung cancer to create a series of gene signatures describing pan-cancer CAF subtypes (pan-CAF) (51). In total they described 5 pan-CAFs, each with distinct gene expression patterns; myofibroblast (myCAF), desmoplastic (dCAF), inflammatory like (iCAF and iCAF-2) and proliferating (pCAF) - each named to represent their predicted function. Applying the newly defined pan-CAF gene sets to bulk RNA sequencing data across a variety of cancer types not only confirmed the presence of multiple CAF subtypes across different tumours, but survival analysis also revealed that in different settings, different pan-CAFs subtypes were predictive of poor outcomes. In bladder cancer, a high infiltrate of the myCAF signature correlated with poor prognosis in The Cancer Genome Atlas (TCGA) dataset, which consists primarily of cystectomy specimens (51).

Studying CAF heterogeneity in breast cancer, Cremasco et al. identified two distinct populations in mouse and human tumours; FAP+ PDPN+ CAFs and FAP+ PDPN- cancer-associated pericytes (CAPs) (52). They showed that FAP+ PDPN+ CAFs, but not CAPs, were capable of supressing T cell proliferation and, *via* deposition of ECM, trapping infiltrating immune cells in the peritumoral regions - a process which appears to be driven by TGFβ (52).

A further key study exploring CAFs subtypes in breast cancer by Costa et al. used flow cytometry with several common CAF markers to reveal the presence of four distinct CAFs (53). These subtypes were differentially expressed in luminal A, triple negative (TNBC) and human epidermal growth factor receptor 2 (HER2)+ breast cancer and showed distinct protein expression signatures and spatial patterns. Two of the identified CAFs were αSMA+ and were classified as CAF-S1 and CAF-S4; each had distinct transcriptomic profiles and pro-tumour functions. Of note, the CAF-S1 (CD29^Med^, FAP^Hi^, fibroblast-specific protein 1 (FSP1)^Low-Hi^, αSMA^Hi^, PDGFRβ^Med-Hi^, caveolin 1 (CAV1)^Low^) subset was associated with an immunosuppressive phenotype *via* the recruitment of Foxp3+ CD4+ CD25+ regulatory T-cells (Tregs) and inhibition of cytotoxic CD8+ T-cells. In contrast CAF-S4 did not play a role in the immune response and instead was found to be associated with muscle contraction, regulation of the actin cytoskeleton and oxidative metabolism (53).

CAF-S1 and CAF-S4 were also identified in high grade serous ovarian cancer where the immunosuppressive function of CAF-S1 was ascribed to the expression of CXCL12β which recruits Tregs *via* CXCR4, this cell-cell contact subsequently enhances survival and differentiation of Tregs (54). CAF-S4 cells on the other hand, did not express CXCL12β, likely due to silencing by micro RNAs miR-141 and miR-200a (54), reinforcing the functional difference between CAF-S1 and CAF-S4.

**Figure 2:**
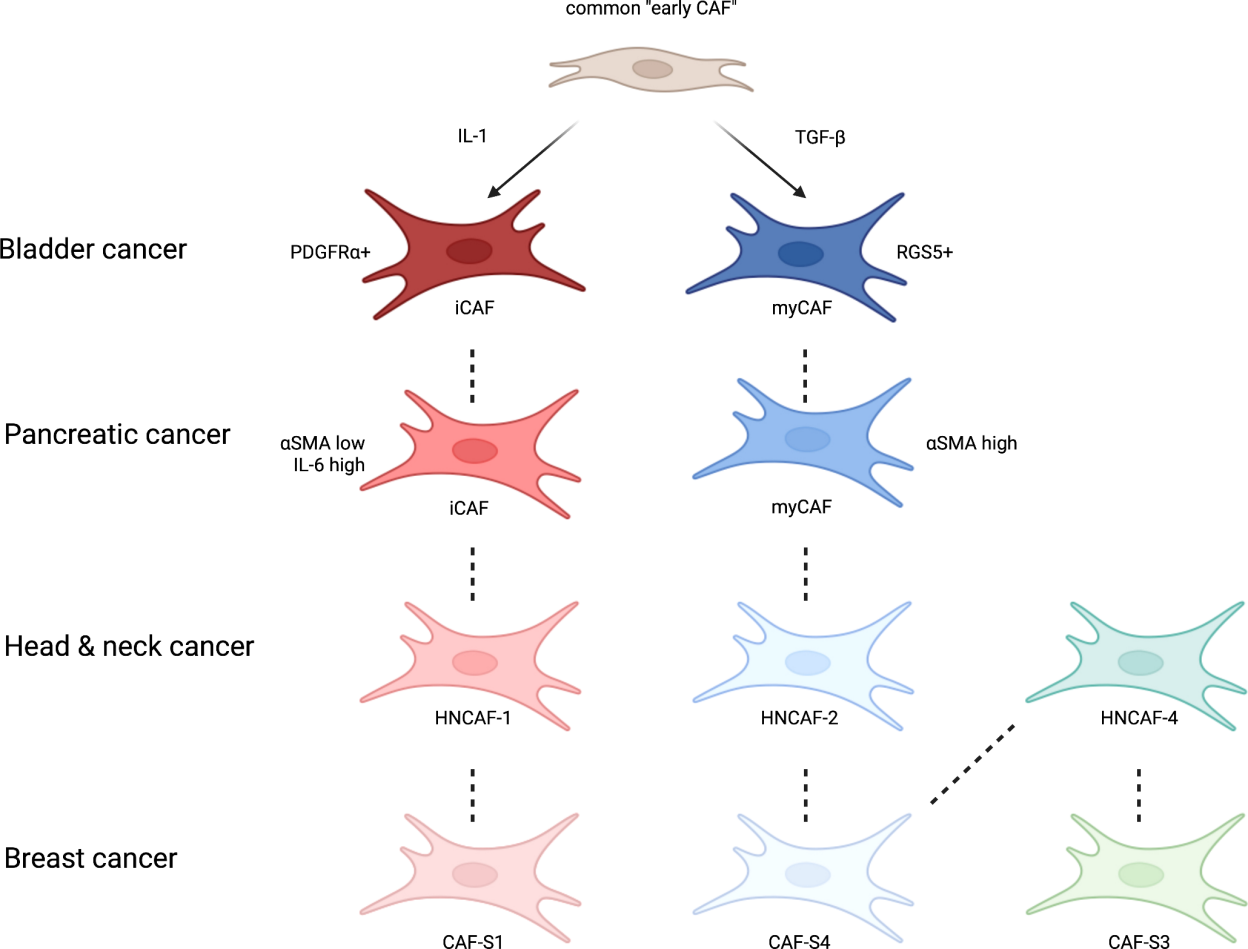
A simplified schematic of CAF subtypes centred around bladder cancer and their similarity with CAFs reported in other common cancers. Derived from a common CAF progenitor (25) and activated by IL-1 and TGFβ (26), in bladder cancer inflammatory CAFs (iCAFs) and myofibroblast CAFs (myCAFs) are delineated by PDGFRα and RGS5 (56). These CAF subtypes and others have been described in other common cancers, such as pancreatic (24), head and neck (55) and breast (53) with their own respective protein markers or transcriptional profiles. Figure adapted from Obradovic et al., 2022. Figure created with Biorender.com.

Obradovic et al. recapitulated Costa’s findings in head and neck squamous cell carcinoma identifying all CAF-S1 to CAF-S4 subsets (55). In addition, they showed that clustering CAFs by flow cytometry lacks resolution and instead inferred protein activity from gene expression data and the enrichment of transcriptional targets using the Virtual Inference of Protein-activity by Enriched Regulon (VIPER) algorithm. In doing so, this added depth to CAF subtype classification and showed that the CAF-S1 subtypes could be further divided into 3 groups with differing effects on response to programmed cell death protein 1 (PD-1) checkpoint inhibition (55). This work highlights potential tissue-specific differences in CAF biology, but, crucially, helps to establish consistency in the results produced by independent groups in different tissue types (**Figure 2**).

In addition to diversity driven by the cell of origin, plasticity in CAF profiles has been shown to change temporally throughout tumour development (57). Not only does the amount of stroma increase with stage, but Elwakeel et al. showed a dramatic increase in the proportion of regulator of G protein signalling 5 (RGS5)+ CAFs, denoted as vasculature CAFs (vCAFs), in the TME of late-stage mouse mammary tumours (57). In contrast, matrix CAFs (mCAFs) were more prevalent in untransformed mammary glands and indeed declined with tumorigenesis. The contribution of a third subset of CAFs was unchanged (57). A temporal shift in CAF biology may be indicative of environmental cues that change within the TME as the tumour progresses. It is therefore of great interest to explore how different therapeutic options can alter signals in the TME and potentially exploit CAF plasticity.

## 3 CAFs in bladder cancer

### 3.1 Bladder cancer-specific CAF heterogeneity – current knowledge

In recent years, with the aid of single cell sequencing technologies, our understanding of the cell populations in the TME of bladder cancer and their respective contributions to disease progression has substantially developed. The application of this technology in bladder cancer is limited to a handful of studies with conservative patient numbers, but, interestingly, findings to date suggest a prominent role for CAFs in bladder cancer biology.

Chen et al. performed scRNAseq on 8 bladder cancer patients and found that the fibroblast population could be stratified into iCAFs or myCAFs by the expression of PDGFRα and RGS5 respectively (56). Comparing differentially expressed genes revealed distinct functions of the two CAF subgroups and suggested that iCAFs are likely to be more pro-tumorigenic *via* functions associated with migration, proliferation, and angiogenesis. Moreover, when evaluated in the TCGA bladder dataset, abundance of iCAFs was associated with poor survival outcomes (56). Wang et al. were also able to segregate fibroblasts in this manner and found RGS5+ myCAFs and PDGFRα+ iCAFs in tissue from healthy, NMIBC, MIBC and metastatic bladder cancer tissue from 13 patients (58) thus corroborating the work by Chen et al.

### 3.2 A potential prognostic/predictive biomarker?

Acknowledging the existence of distinct CAF subtypes, Mesheyeuski et al. studied the expression of several canonical CAF markers (FAP, αSMA, CD90, PDGFRα or PDGFRβ) alone, and in combination, using immunohistochemistry, in a mixed cohort of NMIBC and MIBC, to explore associations with survival (59). Unlike other groups (**Table 1**) that have studied survival in the context of a single marker, none of the CAF markers studied by Mezheyeuski et al. were significantly associated with survival when studied alone. Instead, by combining the CAF markers, they showed statistically significant associations with survival and specifically demonstrated that patients with a FAP dominant stromal score had the shortest 5-year survival. Further emphasising the need to explore multiple components of the TME in combination, Mezheyeuski et al. also found that patients with a CD90 dominant stroma and a high CD8+ T-cell infiltrate had a longer 5-year survival rate (59).

**Table 1:** An overview of research studies that have evaluated individual markers of CAFs in bladder cancer.

Marker	Notes	Method	Tissue type	Ref
PDPN	Patients with over 1% PDPN+ stromal cells had significantly worse RFS following cystectomy. PDPN didn’t predict response to chemotherapy.	IHC	Cystectomy	Okajima et al., 2020 (60)
PDPN	PDPN+ cells found to be infiltrating tumour regions were associated with worse survival. Tumours with less PDPN+ CAFs were likely to have a better response to chemotherapy.	IHC	Cystectomy	Zhou et al., 2020 (61)
CALD1	High CALD1 expression was associated with shorter overall survival in bladder cancer.	TCGA gene expression	Cystectomy	Du et al., 2021 (62)
FAP	When co-expressed with basal markers CK5/6 and CD44, FAP was a strong prognosticator of disease-specific survival in bladder cancer.	IHC	Cystectomy	Calvete et al., 2019 (63)
LRRC15	LRRC15+ myCAFs were associated with poor response to anti-PD-L1 treatment in bladder cancer trial of atezolizumab.	RNAseq	Not stated	Dominguez et al., 2021 (25) Mariathasan et al., 2018 (14)
Kindlin-2	In a study of 203 bladder cancer patients, high Kindlin-2 expression was associated with shorter patient survival.	IHC	Cystectomy and TURBT	Wu et al., 2017 (64)

Podoplanin (PDPN), relapse-free survival (RFS), immunohistochemistry (IHC), Caldesmon 1 (CALD1), Cytokeratin 5/6 (CK5/6), Fibroblast activation protein (FAP), leucine rich repeat containing 15 (LRRC15), single cell RNA sequencing (scRNAseq).

### 3.3 The impact of CAFs on response to treatment

#### 3.3.1 CAFs and the treatment of bladder cancer

Disease progression following bladder-sparing treatments is a real barrier to success in the treatment of bladder cancer. As outlined in the introduction, the survival rates for bladder cancer have remained unchanged for the last five decades. In contrast other common cancers such as prostate and breast cancer have seen large improvements in survival (65).

In metastatic bladder cancer, anti-PD-1/PD-L1 immune checkpoint inhibitors such as nivolumab, pembrolizumab and atezolizumab are routinely used following key phase II and III trials that showed improved overall survival with these agents (66, 67). One of such studies was the CheckMate 275 phase II trial of the anti-PD-1 antibody nivolumab in which biological profiling of tumours was carried out by Wang et al. (15). By exploring stromal parameters in combination with CD8+ T cell infiltration, the study showed that tumours with a high CD8 infiltrate but low stromal scores had the best response to nivolumab. In contrast, a combination of high CD8 infiltrate and high stromal score was detrimental to outcome, and was attributed to an immune excluded phenotype generated by the CAFs and ECM, as discussed above (15).

Having demonstrated the benefits of checkpoint inhibition in the metastatic setting, a separate phase III trial, IMvigor010, considered the use of the anti-PD-L1 monoclonal antibody, atezolizumab, as an adjuvant therapy for patients with high-risk muscle-invasive urothelial carcinoma (68). Despite being generally well tolerated, the trial didn’t reach the intended efficacy endpoints and did not support the use of atezolizumab in this setting (68). However, further investigation of samples produced in the IMvigor010 trial showed that patients with blood samples that were positive for circulating tumour DNA (ctDNA), were better responders to atezolizumab (69). With further validation, ctDNA could help to predict which candidates are likely to respond to adjuvant immune checkpoint inhibition (69). As researchers continue to explore the ways in which immune checkpoint inhibition may improve treatment options for patients with MIBC, perhaps more attention to dual targeting of both CAFs and immune cells would be beneficial.

One of the very few studies to specifically investigate the impact of CAFs on the treatment of bladder cancer found that, of the 28 MIBC patients treated with neo-adjuvant chemotherapy, those with less CAFs in their pre-treatment biopsy were more likely to have a pathological complete response (70). In addition, the tumour cell expression of oestrogen receptor β (ERβ) was lower in tumours with a complete response compared to those with a partial or non-response. Furthermore, the patients with both a higher infiltrate of CAFs and increased expression of ERβ in their post-treatment tumours had a particularly poor response. The same group demonstrated a chemoprotective effect and enhanced proliferation in bladder cancer cell lines when co-cultured with CAFs, versus monoculture, and attributed this effect to the secretion of insulin-like growth factor (IGF) 1 (70).

#### 3.3.2 CAFs in the treatment of other cancers

From the few studies in bladder cancer, it appears that CAFs play a broad role in reducing the efficacy of cancer therapies. However, there is a lack of bladder cancer-specific data on the impact of CAFs on some important therapies including radiotherapy. To expand our understanding of CAFs and their impact on treatments such as radiotherapy, we must look to studies in other cancer settings.

A protective effect of CAFs has been identified in response to radiotherapy for pancreatic cancer. *In vitro* and *in vivo* studies of pancreatic cancer cell lines showed that in monoculture, the growth rate of various pancreatic cancer cells markedly slows following treatment with single dose or fractionated radiotherapy. However, when co-cultured with PSC, the growth rate of pancreatic cancer cells is less affected by irradiation (71). Moreover, co-culturing pancreatic cancer cells with PSC resulted in an increase in the expression of markers associated with EMT and cancer cell stemness induced by TGFβ (72). In a separate *in vivo* study of radiotherapy in PDAC, treatment with radiotherapy resulted in elevated levels of inducible nitric oxide synthase (iNOS) and nitric oxide secreted by CAFs. A combination of radiotherapy with iNOS inhibition delayed PDAC tumour growth in PDAC mouse models (73), which is thought to have occurred because iNOS inhibition reduced CAF-induced pro-tumour effects.

In colorectal cancer, radiotherapy has also been shown to induce the secretion of paracrine factors from CAFs which subsequently enhanced metabolic changes and activation of IGF receptor (IGFR) in neighbouring tumour cells (74). Signalling *via* the IGF1R–Akt–mTOR pathway provides a survival advantage to colorectal tumour cells targeted with radiotherapy. Neutralising IGFR is therefore a potential therapeutic strategy to enhance sensitivity to radiotherapy in patients with CAF-enriched tumours (74).

In rectal cancer, patients with inferior responses to chemoradiotherapy had higher numbers of CAFs in their tumours, and low levels of IL-1 receptor α (IL-1RA) in their serum (75). In searching for the mechanistic basis of these clinical observations, Nicolas et al. showed that reciprocal signalling between CAFs and tumour cells enhanced radiotherapy resistance (76). In an array of studies, they show that IL-1α present in the *ex vivo* culture media of murine tumour cells was responsible for the pre-conditioning of CAFs, resulting in polarisation to an inflammatory iCAF phenotype. Accordingly, they found that increasing levels of nitrite production and signs of oxidative DNA damage made the iCAFs more vulnerable to conversion into a senescent state when treated with irradiation. In this state, iCAFs maintained their capacity to deposit ECM and so continued to create an immune cold and hostile TME that is markedly resistant to therapy. Importantly, reversal of iCAF polarisation *via* IL-1α inhibition seemingly sensitised tumours to radiotherapy and therefore may be a useful combination therapy (76).

Lastly, exploring differences in responder and non-responder breast cancer patients treated with neo-adjuvant chemotherapy, Su et al. found that responders had less CAFs (77). In contrast, the non-responders had a markedly higher CAF population that could be uniquely identified by the surface expression of CD10 and G protein-coupled receptor 77(GPR77). Not only did they find this CAF subtype to be chemoresistant in itself, but the subtype also reduced tumour cell sensitivity to chemotherapy. Furthermore, targeting this CAF subtype with antibodies against GPR77 reversed the chemoresistant effects (77).

Collectively, the evidence reported here suggests that CAFs play a role in the resistance to radiotherapy, chemotherapy, and combinatorial approaches. Likewise, it is apparent that in some cases radiotherapy may exacerbate the pro-tumour CAF phenotype.

## 4 Targeting CAFs – the future of bladder cancer treatment?

The findings discussed earlier in this review indicate that a number of factors are associated with the activation and functionality of distinct CAFs in the TME. This pleiotropic biology, and the lack of a single unifying CAF marker, poses a challenge for therapeutic targeting. However, recent reports have focused on IL-1 and TGFβ.

In this section we will explore the role of TGFβ, a pathway of particular relevance in bladder cancer. We have also indicated a number of studies that have attempted to target CAFs using several strategies in a broad spectrum of different cancers. Although we acknowledge the existence of tumour site specific biology that may affect CAF functionality and expression of phenotypic markers, we also recognise consistent trends throughout the literature that suggest that targeting CAFs in bladder cancer may result in improvements to outcomes, particularly when we consider the impact of CAFs on therapy responses.

### 4.1 The role of TGFβ in bladder cancer

TGFβ has a complex role in the progression of cancer which has been eloquently summarised by Barcellos-Hoff (78). TGFβ has a number of extracellular and intracellular sources, including the ECM which harbours large deposits of latent TGFβ in the form of latency-associated peptide. Under a variety of stimuli, including irradiation of tissue (79), TGFβ is released from the latency complex, resulting in activation of downstream signalling (80). Other TGFβ sources include cancer cells exosomes which can be an important mechanism to activate CAFs (81).

#### 4.1.1 Epithelial mesenchymal transition

In bladder cancer, TGFβ signalling has been shown to be an important stimulus for the induction of EMT (82). Several studies have attempted to define precisely how TGFβ contributes to this outcome and point towards a two-part mechanism whereby TGFβ acts both up and downstream of CAFs.

Comparing the effect of multiple growth factors; TGFβ, acidic fibroblast growth factor (aFGF) and PDGF, Schulte et al. showed that only TGFβ was responsible for the upregulation of markers associated with stromal activation such as αSMA, FSP1 and FAP (40). Furthermore, fibroblasts activated by TGFβ alone, or TGFβ in combination with aFGF, were the most effective at inducing the invasion of RT112 bladder cancer cell lines. Likewise, the combination of TGFβ and aFGF led to a marked increase in the expression of markers associated with EMT (40). To uncover the mechanisms by which CAFs induce EMT, Zhuang et al. showed TGFβ was a vital component of the CAF conditioned media responsible for the induction of EMT in bladder cancer cells (82). They also identified that expression of a long non-coding RNA, ZEB2NAT, in bladder cancer cells, was essential for TGFβ-induced EMT to occur (82).

#### 4.1.2 Immune exclusion

As discussed earlier, TGFβ is a key factor in the stromal exclusion of infiltrating immune cells. Intriguingly, in metastatic bladder cancer, TGFβ signalling status can be used in combination with PD-L1 expression and a score of tumour mutational burden to predict patient responses to the anti-PD-L1 antibody atezolizumab, illustrating how important TGFβ biology is in determining the response to immunotherapy (14). Furthermore, preliminary mouse models, representative of the immune excluded TMEs typically found in metastatic bladder cancer, have been used to demonstrate the effectiveness of TGFβ inhibition in combination with anti-PD-L1. With this combination therapy, a reduction in tumour burden was accompanied by an increase in the number of tumour-infiltrating CD8+ T lymphocytes and crucially such lymphocytes were released from stromal traps to unleash anti-tumour immunity in the appropriate tumour regions (14).

#### 4.1.3 Reprogramming of CAFs

As described earlier, RGS5 has recently emerged as an important marker of the myCAF population in bladder cancer (56). Interestingly, in the pancreas, a high expression of RGS5 on pericytes is typically associated with apoptosis of these cells (83). In contrast, Dasgupta et al. found RGS5+ CAFs in the TME of pancreatic cancers do not undergo apoptosis as expected and instead survive and expand (83). In further exploring this observation, they found TGFβ plays a pivotal role in the reprogramming of CAFs *via* conversion of signalling pathways to allow RGS5 to interact with pSmad2/3 - this resulted in the transcription of genes associated with pro-tumour and proliferative functions (83). If TGFβ is capable of inducing profound reprogramming of RGS5+ CAFs towards a pro-tumour phenotype, it would be interesting to explore how manipulation of TGFβ signalling may affect the RGS5+ myCAF population identified in bladder cancer. We will continue to explore this concept below.

### 4.2 Improvements to therapy responses *via* targeting of CAFs

#### 4.2.1 Manipulation of TGFβ signalling

As we have indicated throughout this review, TGFβ has a fundamental role in the activation and differentiation of CAFs where it typically exerts profound pro-tumour effects. In some cancers, there is evidence that “natural” TGFβ downregulation corresponds with favourable outcomes. This was evident in patients with HPV+ Head and neck squamous cell carcinoma who had less CAFs in their TME and an improved prognosis (84). Wang et al. suggest that miRNA exosomes derived from the tumour cells of these patients were capable of infiltrating CAFs and altering active signalling pathways by reducing the expression of NADPH oxidase (NOX)4 and the presence of reactive oxygen species (ROS). As a result, TGFβ signalling is inhibited (84).

Although the pleiotropic effects of TGFβ make it a challenging target, the above observations encourage further research into novel strategies for manipulation of TGFβ signalling to reduce the pro-tumour impact of CAFs (**Table 2**). It should be noted that TGFβ signaling is important for the regulation of many non-stromal cell types, indeed TGFβ can contribute to both tumour suppression and promotion (91). Therefore targeting TGFβ may result in undesirable consequences in some situations and should be approached with caution. As more of the specific biology associated with TGFβ signalling in CAFs is uncovered, there is hope that a more precise therapeutic approach may become available (25).

**Table 2:** A summary of research studies that have attempted to manipulate TGFβ signalling.

Target	Summary	Stage of development	Ref
TGFβ and PD-L1	Bintrafusp alpha in combination with radiotherapy.	Pre-clinical	Lan et al., 2021 (85)
TGFβR1 and PD-1	MP-VAC-204 trial of Vactosertib with pembrolizumab in metastatic colorectal carcinoma.	Phase Ib/IIa	Kim et al., 2021 (86)
EGFR and SMAD3	Cetuximab in combination with SMAD3 inhibition to downregulate TGFβ in head and neck cancer.	Pre-clinical	Yegodayev et al., 2020 (87)
NOX4/TGFβ axis	GKT137831 reduced ROS in TGFβ-activated CAFs in prostate cancer.	Pre-clinical	Ford et al., 2020 (88)
NOX4	Diphenylene iodonium reduced ROS and slowed bladder cancer cell growth.	Pre-clinical	Shimada et al., 2011 (89)
NOX4	Magnesium isoglycyrrhizinate prevents tumour cell growth in bladder cancer.	Pre-clinical	Yuan at al. 2022 (90)

transforming growth factor β (TGFβ), TGFβ receptor 1 (TGFβR1), programmed death-ligand 1 (PD-L1), epidermal growth factor receptor (EGFR), NADPH oxidase 4 (NOX4), reactive oxygen species (ROS).

One novel method to manipulate TGFβ uses a dual TGFβ and PD-L1 targeting approach with a novel fusion protein called bintrafusp alpha. In combination with radiotherapy, bintrafusp alpha proved effective at reducing the fibrotic networks induced by TGFβ-activated CAFs, and increased the infiltration of cytotoxic CD8+ T-cells in multiple immunocompetent mouse modes of PDAC, glioblastoma, neuroendocrine colonic carcinoma and mouse mammary carcinoma (85).

Similar findings have been observed in the phase Ib/IIa MP-VAC-204 trial which involved a combination of the TGFβ receptor type 1 inhibitor Vactosertib with the PD-1 inhibitor pembrolizumab to treat patients with microsatellite stable metastatic colorectal carcinoma (mCRC) (86). The initial results of MP-VAC-204 are promising and show a decrease in biomarkers of TGFβ signalling and crucially an increase in CD8+ T-cell infiltration. Where previously pembrolizumab was effective for a small percentage of patients with microsatellite instability mCRC, the addition of TGFβ inhibition appears to increase the number of patients who may benefit from checkpoint inhibition (86).

Taking a different approach, Yegodayev et al. found a stromal increase in TGFβ signalling to be associated with a poor response to the epidermal growth factor receptor (EGFR) antibody cetuximab in patient-derived xenograft models of head and neck cancers (87). Moreover, cetuximab efficacy could be greatly improved with the combined use of a SMAD3 inhibitor to block the downstream effects of TGFβ (87).

Promising strategies to target the NOX4/TGFβ axis have also been explored. They include the NOX1/NOX4 inhibitor GKT137831, which reduced the production of ROS in TGFβ-activated CAFs in prostate cancer and further dissipated the pro-tumour functions of CAFs (92). In a number of mouse models, GKT137831 “normalised” CAFs and, as a result, stranded CD8+ T-cells were able to infiltrate tumours and were more responsive to immunotherapeutic agents such as anti-PD-1 (88). Similarly, *in vitro* studies of bladder cancer have demonstrated the efficacy of the NOX4 inhibitor diphenylene iodonium, which slowed cancer cell growth *via* a reduction in the expression of ROS (89). Lastly, magnesium isoglycyrrhizinate has also been tested *in vitro* and *in vivo* in the bladder cancer setting and showed promise as a potential NOX4 inhibitor that prevents tumour growth (90).

#### 4.2.2 Alternative strategies to target CAFs

Strategies to combine CAF targeting agents other than TGFβ inhibitors with immune checkpoint blockade have also shown pre-clinical promise. Nintedanib is a tyrosine kinase inhibitor which has anti-fibrotic effects in the TME and contributes to an anti-tumour response *via* the release of previously excluded immune cells (93). The addition of anti-PD-1 treatment enhanced responses to treatment with nintedanib and slowed the growth of B16-F10 melanoma tumours *in vivo* (93). Intriguingly, nintedanib has recently shown promise when combined with neo-adjuvant chemotherapy in localised MIBC. In the NEOBLADE trial, addition of nintedanib to standard radical treatment significantly improved overall survival, suggesting this drug warrants further consideration in bladder cancer (94).

Alternative attempts to target CAFs, and therefore slow the progression of cancer, have included the use of FAP-specific chimeric antigen receptor (CAR) T-cells. *In vitro* and *in vivo* studies have demonstrated the feasibility of targeting CAFs using this approach, and show early signs of promise that FAP-specific CAR T-cells can reduce tumour burden (95, 96). However, by targeting CAFs we have continued to learn about crucial elements of their biology. Several attempts to deplete the fibroblast component of the TME and the associated ECM have unfortunately resulted in enhanced tumour progression. This finding not only supports the notion that CAFs are a heterogenous population, but that CAFs can also function in a manner that restrains tumour growth in specific contexts. Although we do not wish to over-emphasize this point, it is important we include this for completion.

One such example was demonstrated in a genetically modified mouse model of PDAC in which αSMA+ myofibroblasts could be depleted at a given time point in a drug dependent manner. Depletion at both early and late stages of PDAC development resulted in reorganisation of the associated ECM, a reduction in infiltrating effector T-cells, and an increase in regulatory T-cells, all of which combined to create significantly worse survival outcomes compared to control mice (97).

Similarly, progression of PDAC tumours was observed when the stromal driving factor sonic hedgehog (Shh) was deleted from tumour cells. Under these conditions, the PDAC TME contained less stromal cells, but was more vascular and proliferative (98). Deletion of stromal components such as type one collagen, a crucial part of the ECM, had a similar effect (99).

Although there have been very few studies targeting CAFs in bladder cancer, we can apply our knowledge of bladder cancer biology to infer similarities with tumour types such as PDAC (97). For example, Shin et al. showed that in mouse and human tumours loss of Shh was associated with progression from carcinoma *in situ* to MIBC (100). As such tumour cells were no longer able to induce stromal cell differentiation *via* Shh, this resulted in a proliferative tumour lacking restraint from neighbouring stromal cells (100).

Rather than deplete specific CAF populations, a new trial reported by Mizutani et al. considered the plasticity of CAFs and proposed a new treatment regimen for patients with advanced pancreatic cancer that could convert tumour-promoting CAFs (pCAFs) into tumour-restraining CAFs (rCAFs) *via* the addition of AM80 (101). In combination with chemotherapy, AM80, a synthetic retinoid, converted pCAFs into rCAFs marked by the upregulation of Meflin and downregulation of the canonical CAF marker αSMA. Although this phase I trial is in the initial stages, it offers an insight into the potential direction of research into CAFs and the future strategies that may be undertaken to target them (101).

In a similar manner, manipulation and reversal of CAF activation has been attempted using Vitamin D (102, 103). Indeed, in colorectal carcinoma (CRC) an increase in the expression of Vitamin D receptors (VDR) on fibroblasts in the TME was associated with improved survival outcomes (102). Ferrer-Mayorga et al. subsequently found that an increase in the active form of Vitamin D (1,25(OH)_2_D_3_) prevented activation of normal fibroblasts into CAFs and induced a gene signature that was associated with improved survival outcomes in CRC patients (102). Experiments using the VDR ligand calcipotriol had a similar effect in PDAC (103).

Understanding CAF biology not only helps to develop complementary treatment options, but it can also help to optimise current ones. One CAF targeting strategy takes an alternative approach and uses a 68Ga-radiolabeled inhibitor of FAP (FAPI) to visualise CAFs on PET/CT scans for patients with head and neck squamous cell carcinoma (104). In this novel study, visualisation of FAP+ CAFs acts as both a diagnostic tool and a therapeutic guide helping to risk stratify patients and improve the delivery of radiotherapy (104).

## 5 Conclusion

This review has discussed how further research into the role of CAFs in bladder cancer is necessary to fill important gaps in our knowledge of this disease and expand therapeutic options for patients. Additionally, it is particularly important to understand how CAFs impact the response to therapies currently available to patients. Based on the evidence presented in this review, we hypothesise that CAF enrichment of bladder tumours is associated with inferior responses to radical radiotherapy for MIBC. Poor responses to radiotherapy are seen in other tumour types with a high stromal score and an increased number of CAFs, such as rectal cancer (75). If, as we hypothesize, MIBC enriched for CAFs show similar behavior, this highlights a cohort of patients that we propose would benefit from a combinatorial approach that includes targeting of CAFs. In addition, CAF targeting approaches may have a role in metastatic disease. In the United Kingdom, the RE-ARM trial is exploring the combination of immune checkpoint blockade with “experimental” palliative radiotherapy in patients with metastatic urothelial cancer (105). The clinical outcomes of this trial, including the comprehensive translational profiling that is embedded in RE-ARM, may help to identify groups of patients that could benefit from CAF targeting approaches. In doing so, this could expand the number of patients that ultimately benefit from immune checkpoint inhibition.

In conclusion, we propose that the various strategies to target CAFs discussed in this review have considerable therapeutic potential, both as single agents, and in combination with existing therapies, to improve survival outcomes for patients with bladder cancer. In order to inform treatment advances, we consider that it is a research priority to better understand bladder cancer-specific CAF subtypes and their spatial relationship with other cells of the TME at baseline, as well as longitudinal changes in CAF biology following treatments such as radiotherapy.

## Author contributions

AB wrote and edited the manuscript and created the figures. AW proposed the conception, edited and reviewed the manuscript. AR edited and reviewed the manuscript. All authors listed in the paper have made a substantial, direct, and intellectual contribution to the work and approved it for publication. All authors contributed to the article and approved the submitted version.

## Funding

The PhD studentship awarded to AB is supported by the MRC iCASE Doctoral Training Partnership in collaboration with AstraZeneca. Award number: MR/R01583X/1. We acknowledge NHS funding to the NIHR Biomedical Research Centre at The Royal Marsden and the ICR. AW acknowledges funding from the ICR/RMH CRUK RadNet centre.

## Acknowledgments

This study represents independent research supported by the National Institute for Health and Care Research (NIHR) Biomedical Research Centre at The Royal Marsden NHS Foundation Trust and the Institute of Cancer Research, London. The views expressed are those of the author(s) and not necessarily those of the NIHR or the Department of Health and Social Care. AW acknowledges funding from the RMH/ICR Cancer Research UK RadNet Centre.

## Conflict of interest

AB declares funding from AstraZeneca to support the work of their PhD project. AW declares potential funding from imCORE for translational profiling in the RE-ARM study which is sponsored by Roche.

The remaining authors declare that the research was conducted in the absence of any commercial or financial relationships that could be construed as a potential conflict of interest.

## Publisher’s note

All claims expressed in this article are solely those of the authors and do not necessarily represent those of their affiliated organizations, or those of the publisher, the editors and the reviewers. Any product that may be evaluated in this article, or claim that may be made by its manufacturer, is not guaranteed or endorsed by the publisher.
